# Temperature‐sensitive hydrogel releasing pectolinarin facilitate scarless wound healing

**DOI:** 10.1111/jcmm.18130

**Published:** 2024-02-08

**Authors:** Xiaohang Chen, Haoyue Song, Kun Song, Yuan Zhang, Jia Wang, Jinjia Hong, Qingpeng Xie, Jing Zhao, Meixian Liu, Xing Wang

**Affiliations:** ^1^ Shanxi Medical University School and Hospital of Stomatology Taiyuan China; ^2^ Shanxi Province Key Laboratory of Oral Diseases Prevention and New Materials Taiyuan China; ^3^ Department of Oral and Maxillofacial Surgery, The First Affiliated Hospital, Laboratory of Facial Plastic and Reconstruction Fujian Medical University Fuzhou China

## Abstract

The dressing that promotes scarless healing is essential for both normal function and aesthetics after a wound. With a deeper understanding of the mechanisms involved in scar formation during the wound healing process, the ideal dressing becomes clearer and more promising. For instance, the yes‐associated transcriptional regulator (YAP) has been extensively studied as a key gene involved in regulating scar formation. However, there has been limited attention given to pectolinarin, a natural flavonoid that may exhibit strong binding affinity to YAP, in the context of scarless healing. In this study, we successfully developed a temperature‐sensitive Pluronic@F‐127 hydrogel as a platform for delivering pectolinarin to promote scarless wound healing. The bioactive pectolinarin was released from the hydrogel, effectively enhancing endothelial cell migration, proliferation and the expression of angiogenesis‐related genes. Additionally, a concentration of 20 μg/mL of pectolinarin demonstrated remarkable antioxidant ability, capable of counteracting the detrimental effects of reactive oxygen species (ROS). Our results from rat wound healing models demonstrated that the hydrogel accelerated wound healing, promoting re‐epithelialization and facilitating skin appendage regeneration. Furthermore, we discovered that a concentration of 50 μg/mL of pectolinarin incorporated to the hydrogel exhibited the most favourable outcomes in terms of promoting wound healing and minimizing scar formation. Overall, our study highlights that the significant potential of locally released pectolinarin might substantially inhibit YAP and promoting scarless wound healing.

## 1 INTRODUCTION

Skin serves as the primary barrier against injury and is the most vulnerable tissue.^1^ Every year, millions of individuals suffer from surgical trauma or burns, resulting in scars that cause significant physical and psychological damage. In the United States alone, the annual cost of treating skin damage is estimated to be around $50 billion, and the global scar therapy market is projected to reach $35 billion by 2023.^2^, ^3^ Clinical treatments for skin injuries include the use of dressings, skin grafts and pharmacological therapies.^4^ While skin grafts are considered the gold standard for treating deep skin injuries, but the limited availability of donor skin and associated surgical discomfort restrict their widespread use. On the contrary, conventional dressings lack the ability to induce successful scarless healing.^5^ Although current hormonal drugs, such as corticosteroids, can aid in scar removal, their side effects, including osteoporosis and pain, must be taken into consideration.^6^ To expedite scarless wound healing and promote cell proliferation, migration and angiogenesis, a combination of advanced biomaterials and induction factors is required.

Empirical and anecdotal evidence supports the potential of Cirsium japonicum in scarless wound healing, although limited research has been conducted in this specific field.^7^ Based on our unpublished research, we have observed Cirsium japonicum can enhance scarless healing of full‐thickness skin defects in rat models. Further investigation suggests that pectolinarin, a flavonoid compound, may serve as the key active component in Cirsium japonicum responsible for promoting scarless regeneration.^8^ Pectolinarin has garnered increasing attention due to its hemostatic, anti‐inflammatory, antibacterial, antiviral and other pharmacological activities, and has been utilized in the treatment of various conditions such as diabetes and tumours.^9^, ^10^, ^11^ Moreover, pectolinarin has shown promise in the field of tissue repair, where its antioxidant properties have been beneficial in treating liver damage.^12^ However, there is a paucity of studies specifically focused on investigating its potential scarless effects.

The yes‐associated transcriptional regulator (YAP) plays a crucial role as a mediator of the Hippo signalling pathway, contributing to tissue regeneration and repair.^13^ Scar formation is influenced by the presence of Engrailed‐1 (En1)‐positive fibroblasts during the wound healing process, with YAP acting as an upstream gene of En1 that is activated in response to wound tension.^14^ Previous studies have demonstrated the reliability of molecular docking experiments^15^ in predicting intermolecular binding affinity. We found pectolinarin exhibit strongly binding affinity to YAP by molecular docking experiments. However, pectolinarin's insolubility in water poses challenges, as it is susceptible to dilution or loss upon topical application. PF‐127(Pluronic@F‐127), also known as Poloxamer 407, is a well‐known amphiphilic polymer with characteristic such as low toxicity, heat reversibility and biocompatibility.^16^, ^17^, ^18^ Furthermore, these thermosensitive polymer facilitate the solubilization of hydrophobic drugs while controlling release rates to avoid rapid release.^19^ They have found wide applications in cosmetics, eyeglass cleaning solutions,^18^, ^20^ and as delivery platforms for exosomes, medicines, cells and other biologically active substances to promote local tissue regeneration.^21^, ^22^, ^23^, ^24^

To facilitate scarless regeneration, we developed a composite dressing by incorporating pectolinarin into PF‐127 hydrogels. Our focus was on evaluating the dressing's ability to promote vasculature regeneration, a crucial process in wound healing. Using a rat full‐thickness skin defects model, we observed that the dressing effectively enhance wound healing and minimized scar formation, particularly at a pectolinarin concentration of 50 μg/mL. Additionally, we conducted an experiment to assess the dressing's antioxidant properties by measuring reactive oxygen species. These findings contribute to the advancement of novel biomaterials for scarless regeneration.

## 2 METHODS AND MATERIALS

### 2.1 Cell culture

L‐929 fibroblasts and EA. hy926 endothelial cells were obtained from the College of Materials Science and Engineering at Taiyuan University of Technology. The cells were cultured in Dulbecco's Modified Eagle Medium (DMEM) supplemented with 10% fetal bovine serum (FBS) and 1%penicillin/streptomycin at 37°C with 5% CO_2_. When the cells reached 80% confluence, they were passaged using trypsin.

### 2.2 Preparation of PF‐127@Pectolinarin hydrogels

A 30% PF‐127 (Sigma‐Aldrich, USA) solution^25^ was prepared and filtered through a 0.22 μm membrane. To evaluate the coagulation of the hydrogel, 2 mL of the PF‐127 solution was poured into a 5 mL glass container and incubated in a 37°C water bath. The temperature sensitivity of the hydrogel was assessed by storing the coagulated hydrogel at 4°C. PF‐127 solutions containing pectolinarin (MedChemExpress, China) at concentrations of 0, 25, 50, 100 and 200 μg/mL were prepared in an ice bath, and the gel performance was evaluated.

### 2.3 Evaluation of pectolinarin release performance

The maximum absorption wavelength of pectolinarin was determined using an ultraviolet–visible spectrophotometer.^26^ A standard curve (S1A) was drawn using the absorbance value of a pectolinarin solution at various concentrations at the maximum absorption wavelength was 332 nm. PF‐127 hydrogels containing 0, 25, 50, 100 and 200 μg/mL pectolinarin were prepared and defoamed overnight at 4°C. The hydrogels were then allowed to solidify at room temperature for 30 min before adding 9 mL of PBS (pH 7.4) and incubating at 37°C with 100 rpm agitation. Pectolinarin release was measured by removing 100 μL of supernatant at 30 min, 1 h, 3 h, 6 h, 12 h, 24 h and 36 h and measuring the absorbance value at the maximum absorption wavelength. The experiment was repeated three times.

### 2.4 Production and testing of reactive oxygen species in cells

The level of reactive oxygen species in cells was detected using DCFH‐DA fluorescent probes.^27^ L‐929 cells were treated with different concentrations of H_2_O_2_ (0, 10, 25, 50 and 100 μM) as the control group, and the experimental group was treated with pectolinarin (20 μg/mL) combined with H_2_O_2_ at the same concentrations. After 2 h of treatment, the cells were washed in serum‐free media and incubated with 10 μM DCFH‐DA (Beyotime, China) for 30 min, followed by 5 min with Hoechst33342 staining solution. Fluorescence expression was examined using a fluorescent microscope and analysed using Image J.

### 2.5 Cell live and death assay and morphological analysis

Cell viability was determined using the Calcein‐AM/PI staining assay.^28^ L‐929 cells were inoculated onto a 24‐well plate and treated with different extracts of PF‐127@P hydrogels (ISO 10993‐12:2021) on Day 1 and Day 3. Live cells were stained with diluted Calcein‐AM dye for 20 min, and dead cells were stained with diluted PI dye for 10 min before being visualized under a fluorescence microscope. Phalloidin dye (MedChemExpress, China) was used to detect cell morphology. Cells were fixed for 40 min in 4% paraformaldehyde, stained with phalloidin for 30 min to visualize the cytoskeleton, and stained with DAPI for 5 min to visualize nuclei before being examined under a fluorescence microscope.

### 2.6 RT‐qPCR

The total RNA of the cells was collected after 24 h of coculture with different PF‐127@P hydrogel extracts, and the RNA was transformed into cDNA using the ReverTra Ace qPCR RT kit (Toyobo, Japan). Gene expression was measured using the SYBR green kit (Mei5bio, China) with an amplification programme of 95°C for 30 s, followed by 40 cycles of 95°C for 15 s, 60°C for 15 s and 72°C for 30 s. GAPDH was used as an endogenous control, and the relative gene expression was calculated using the 2^−ΔΔCT^ method. Data were analysed using GraphPad Prism, and each experiment was repeated three times. The sequences of all primers used in the study are listed in Table 1.

**TABLE 1:** The sequence of all primers in the study.

Gene	Primer
Human‐GAPDH‐ FORWARD	5′‐CGGAGTCAACGGATTTGGTCGTAT‐3′
Human‐GAPDH‐ REVERSE	5′‐AGCCTTCTCCATGGTGGTGAAGAC‐3′
Human‐vWF‐FORWARD	5′‐TGCGACACCATTGCTGCCTATG‐3′
Human‐vWF‐REVERSE	5′‐GCCACTCACACTCATACCCGTTC‐3′
Human‐ANG‐1‐FORWARD	5′‐CTCCTTGGAGAAGAGTTACGAG‐3′
Human‐ANG‐1‐REVERSE	5′‐TCACACTTCATGATGGAGTTGA‐3′
Human‐eNOS‐FORWARD	5′‐AGCATATGGACTCAGACAGTTC‐3′
Human‐eNOS‐REVERSE	5′‐CAGACATTCTGGACATCTACCA‐3′
Human‐VEGFA‐FORWARD	5′‐AGAAGGAGGAGGGCAGAATCATCAC‐3′
Human‐VEGFA‐REVERSE	5′‐GGGCACACAGGATGGCTTGAAG‐3′
Mouse‐GAPDH‐FORWARD	5′‐TGACCACAGTCCATGCCATC‐3′
Mouse‐GAPDH‐REVERSE	5′‐GACGGACACATTGGGGGTAG‐3′
Mouse‐YAP‐FORWARD	5′‐TGACAACCAATAGTTCCGATCCCTTTC‐3′
Mouse‐YAP‐REVERSE	5′‐CCACACTGTTGAGGAAGTCGTCTG‐3′
Mouse‐En1‐FORWARD	5′‐CAAGCGTGCCAAGATCAAGAAAGC‐3′
Mouse‐En1‐REVERSE	5′‐TGTCCTGAACCGTGGTGGTAGAG‐3′
Mouse‐α‐SMA‐FORWARD	5′‐TGGCCACTGCTGCTTCCTCTTCTT‐3′
Mouse‐α‐SMA‐REVERSE	5′‐GGGGCCAGCTTCGTCATACTCCT‐3′

### 2.7 CCK8 assay

Endothelial cells were inoculated in a 96‐well plate at a density of 1 × 10^4^ cells per well and cultured in regular medium for 1 day. The medium was then changed to complete medium containing PF‐127, PF‐127@P25, PF‐127@P50, PF‐127@P100 and PF‐127@P200 extracts, while the blank control group used PBS. Each group had three replicate wells. At 24 h, 48 h and 72 h, CCK8 solution (MedChemExpress, China) was added, and the absorbance value was measured at 450 nm using a microplate reader. Statistical analysis was performed using GraphPad Prism.

### 2.8 Scratch test

Endothelial cells were inoculated in 6‐well plates and cultured in regular medium. Once the cells reached 120% confluence, the plates were vertically scratched with a sterile 200 μL tip and washed with PBS until the cell debris was removed. The serum‐free medium containing PF‐127, PF‐127@P25, PF‐127@P50, PF‐127@P100 and PF‐127@P200 extracts was added, and the plates were incubated. Images were captured under a microscope at 0 h, 12 h and 24 h, and the data were analysed using Image J.

### 2.9 Hemolysis test

2 mL of fresh blood was drawn from the rat's jugular vein, centrifuged at 1500 rpm for 10 min, and the blood cell pellet was washed with PBS three times until the supernatant became clear. The washed red blood cells were then resuspended in PBS to a concentration of 2% (v/v). PF‐127, PF‐127@P25, PF‐127@P50, PF‐127@P100 and PF‐127@P200 extracts were added to the red blood cells, and the mixtures were incubated at 37°C for 1 h. Subsequently, the mixtures were centrifuged at 1500 rpm for 5 min, and the supernatant was collected to measure the absorbance at 540 nm using a microplate reader. The positive control group used distilled water, and the negative control group used PBS. The hemolysis rate was calculated using the following formula:


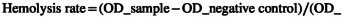
 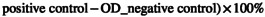


The hemolysis rate was classified as follows: <5% as nonhemolytic, 5%–10% as slightly hemolytic, 10%–20% as moderately hemolytic and >20% as highly hemolytic.^29^

### 2.10 Establish a skin defect model

All animal experiments in this study were approved by the Ethics Committee of Shanxi Medical University (Approval ID: 2021–207). Thirty male Sprague Dawley (SD) rats weighing 130 g ± 10 g and aged 4 weeks were purchased and allowed to adapt to their environment for 1 week prior to the experiment. Rats were randomly assigned to six groups: Control, PF‐127, PF‐127@P25, PF‐127@P50, PF‐127@P100 and PF‐127@P200 dressing group. The rats were anaesthetised with 10% chloral hydrate (0.3 mL/kg), the hair on their backs was shaved with a razor, and the skin was disinfected with iodophor. A 1 cm circular area was marked and the full‐thickness skin was excised using ophthalmic scissors. In order to avoid secondary trauma such as additional surgeries and anaesthesia, we replenished an adequate amount of gel to ensure coverage of the wound every 3 days. Wound healing was measured and photographed on days 0, 3, 7, 10 and 14, and the weight was recorded. The wound size was quantified using ImageJ analysis software to calculate the wound healing rate.^30^ On day 14, the rats were euthanized, and the wound and surrounding skin were excised and fixed in 4% paraformaldehyde for further use.

### 2.11 Haematoxylin and eosin staining and Masson staining

The fixed specimens were placed in an embedding box, rinsed with running water to remove excess fixative and dehydrated with alcohol gradients. Subsequently, the specimens were soaked in xylene to remove any remaining alcohol, embedded in wax, sliced, baked and pasted onto glass slides before being dried. Before performing haematoxylin and eosin or Masson staining, the slides were dewaxed again and soaked in a series of alcohol concentrations from high to low, followed by a final rinse in distilled water.

For haematoxylin and eosin staining, the slides were first immersed in an aqueous solution of haematoxylin, followed by rinsing with acid water and ammonia water for 1 h. The slides were then immersed in distilled water, dehydrated with alcohol, and finally stained with eosin staining solution. The stained sections were dehydrated, cleared, mounted and observed.

Masson staining was performed according to the manufacturer's instructions. The glass slides were wetted with distilled water, and the nuclear and plasma dyes were applied for 1 min each before being flushed with running water for 1 min. Then, the colour separation solution was applied for 8 min, followed by the negative dye solution for 5 min. The slides were rinsed with absolute ethanol, mounted and observed.

### 2.12 Immunohistochemistry (IHC)

The slides were baked, dewaxed and rehydrated before being heated in a sodium citrate buffer solution, and then allowed to cool naturally. The tissue blocks were marked, and the slides were rinsed with PBS. To block endogenous peroxidase activity, 3% H_2_O_2_ was added and incubated for 10 min, followed by a rinse with PBS. BSA was added to neutralize peroxide and incubated for 10 min before adding the primary antibody. After washing, the slides were incubated overnight at 4°C. The slides were rinsed with PBS, and then the secondary antibody was added and incubated with horseradish peroxidase for 1 h. After washing, the colour developing agent was added, and the slides were rinsed after colour development was completed. Finally, the slides were negatively stained with haematoxylin and mounted.

### 2.13 Statistical analysis

Image and data processing and analysis were mostly done using ImageJ and GraphPad Prism 8, respectively. All data were expressed as mean ± standard deviation, and one‐way ANOVA was used for comparison between groups, while two‐tailed Student's *t*‐test were used when compared paired samples and Tukey's test were used when there were multiple independent groups. A *p*‐value of less than 0.05 indicated a significant difference, represented by an asterisk in the figure. Each experiment was repeated at least three times.

## 3 RESULTS AND DISCUSSION

### 3.1 Construction and characterization of PF‐127@P dressings

**FIGURE 1:**
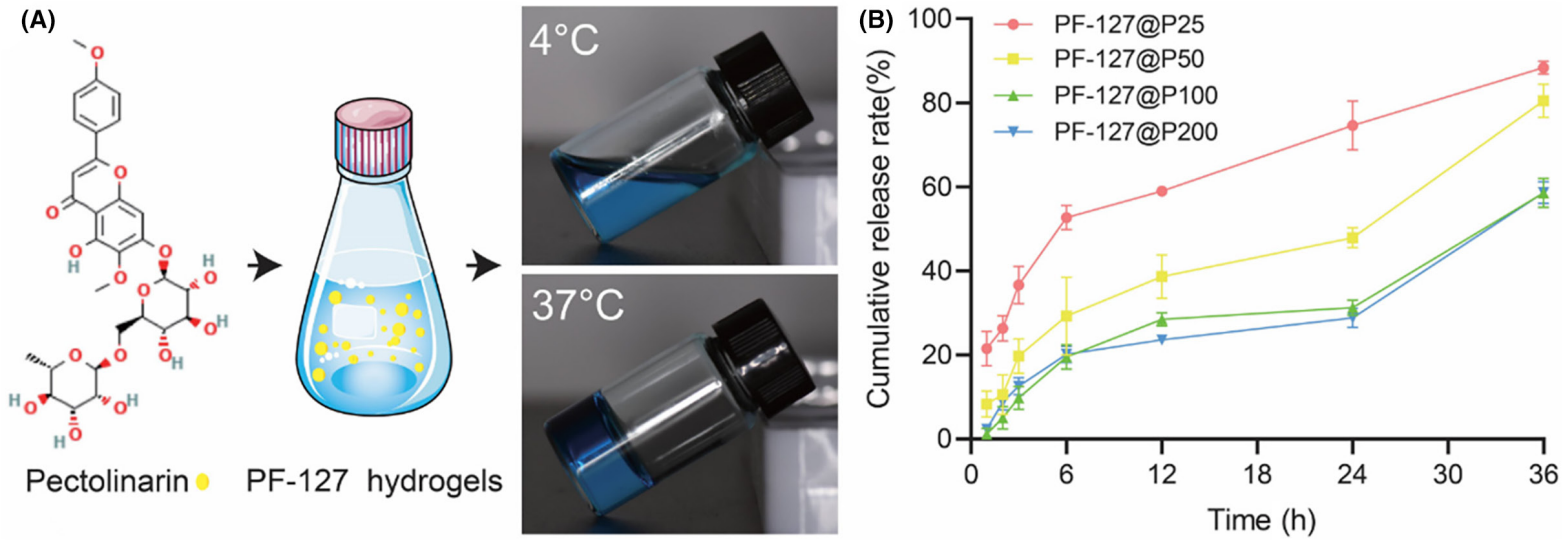
The construction and characterization of PF‐127@P dressings. (A) Image of PF‐127@P hydrogels at 4°C and 37°C. (B) Cumulative release rate (%) of PF‐127@P hydrogels containing different concentrations of pectolinarin at 1, 2, 3, 6, 12, 24 and 36 h in vitro.

We fabricated 30% PF‐127 solution that remains in a liquid state at 4°C but transitions to a gelatinous state at 37°C (Figure 1A). This property enables easy application of the solution to irregular wounds. PF‐127 is an amphiphilic polymer known for its ability to enhance drug solubility. In fact, it can increase the solubility of the insoluble medicine fenofibrate in PF‐127 solution by up to 45,000 times compared to hydrophilic solutions.^31^, ^32^ Due to its hydrophobic nature, PF‐127 can also assist in the release of insoluble medicines. As a result, PF‐127 can serve as a carrier for pectolinarin, significantly increasing its solubility.

We found that the setting time of the hydrogel was unaffected by the addition of pectolinarin. They both turn into a gel within 1 min at 37°C. To evaluate the sustained release of pectolinarin from the PF‐127 hydrogel, we conducted experiments at various time points following in a 37°C shaker (speed = 100 rpm). Surprisingly, we observed that pectolinarin did not burst from the hydrogel, as shown in Figure 1B. As the pectolinarin loading increased, the cumulative release rate of pectolinarin decreased. After 36 h, the total drug release of the PF‐127@P25 and PF‐127@P50 reached 80%, while the sustained release of the PF‐127@P100 and PF‐127@P200 was around 50%. These findings indicate that higher pectolinarin loading leads to longer release times. Previous studies have suggested that the maximum drug loading capacity of PF‐127 is around 0.5% (w/w), and beyond this threshold, a sudden release occurred. In our study, the concentration of 200 μg/mL did not exceed this threshold.^33^

Considering the potential adverse effects of excessive pectolinarin release, we investigated its impact on rheumatoid arthritis fibroblast‐like synoviocytes. At a concentration of 20 μM, pectolinarin inhibited cell proliferation, induced apoptosis and reduced inflammation.^34^ Furthermore, pectolinarin has been shown to have no substantial inhibitory effect on neuroblastoma and eosinophil cell at concentrations below 100 μg/mL.^35^ There are many hydrogels or nanomaterials to choose from as drug‐carrying systems, and in this study, which focuses on the pro‐wound healing and antiscarring effects of pectolinarin, we only investigated whether the drug could be slow‐released out of the PF‐127 hydrogel.

### 3.2 Biocompatibility of PF‐127@P dressings

#### 3.2.1 Cytobiocompatibility

**FIGURE 2:**
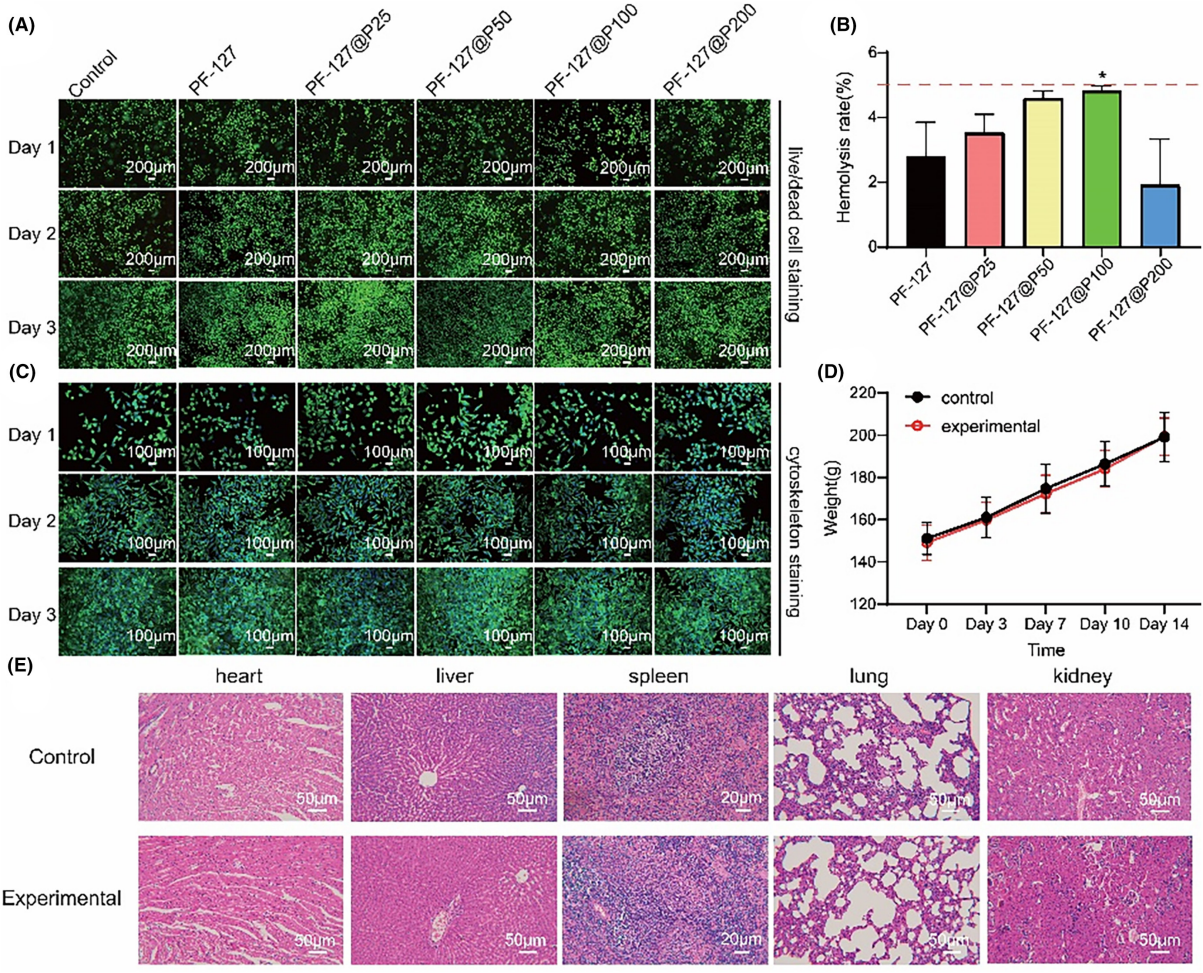
Biocompatibility of PF‐127@P dressings. (A) Live/dead staining of coculture with dressing's extract for 1, 2 and 3 days, red is dead cells and green is live cells. (B) Hemolysis rate. *p < 0.05 when compared with PF‐127. (C) Cytoskeleton staining of coculture with dressing's extract for 1, 2 and 3 days, blue is nucleus and green is cytoskeleton. (D) Changes in body weight of experimental group (red, hydrogel wiping group) and blank group (black). (E) Major organ changes in rats with dressing (experimental) and control group.

Biocompatibility is a crucial factor for the medical application of materials. To assess the cytocompatibility of PF‐127@P hydrogels, cells were inoculated on cell culture plates and stained with calcein‐AM/PI to observe live/dead cells (Figure 2A). Additionally, phalloidin staining was used to visualize changes in cytoskeleton morphology (Figure 2C). On day 1, the cells appeared rounder, indicating limited spreading. Only the PF‐127@P200 group showed slightly higher red fluorescence in the live/dead cell staining on day 2, which might be attributed to cytotoxicity from excess pectolinarin. However, this difference was not statistically significant compared to other groups, suggesting good cytocompatibility of the dressing.

#### 3.2.2 Hemocompatibility

In the context of skin defect repair, it is crucial to evaluate whether materials have any toxic effects on blood locally. Hemolysis testing was performed to assess the hemocompatibility of PF‐127@P hydrogels. Blood cells were incubated with the hydrogels for 1 h, and the hemolysis rates were measured. As depicted in Figure S1B, there was no visible hemolysis observed between the hydrogels and the red blood cells, indicating good hemocompatibility. The hemolysis rates of the materials were all below 5%.

#### 3.2.3 In vivo biocompatibility

The in vivo toxicity of PF‐127@P dressings was evaluated in rats. Vital signs and behaviour of the rats were monitored and recorded throughout the 14‐day wound healing process, with no significant changes observed. The rats' body weight increased steadily over time (Figure 2D). Haematoxylin and eosin staining of heart, liver, spleen, lung and kidney tissues from both the experimental and control groups (Figure 2E) revealed no damage, such as inflammation and abnormal neovascularization, caused by the use of PF‐127@P dressings. These results indicate the excellent biocompatibility of the dressing.

### 3.3 Antioxidant ability of pectolinarin

Reactive oxygen species (ROS), natural byproduct of oxygen metabolism, play important roles in regulating cell proliferation, differentiation, tissue damage repair and immune responses under normal physiological conditions.^36^, ^37^ However, excessive ROS can cause damage to proteins, nucleic acids and lipids in cells, inhibiting endothelial cell and fibroblast proliferation, migration and differentiation, and even leading to cell necrosis. This creates an unfavourable microenvironment for wound healing.^38^, ^39^ Pectolinarin, a flavonoid and natural antioxidant, acts by inhibiting the activation of enzymes associated with free radicals production through the superoxide dismutases (SOD) antioxidant mechanism.^40^

**FIGURE 3:**
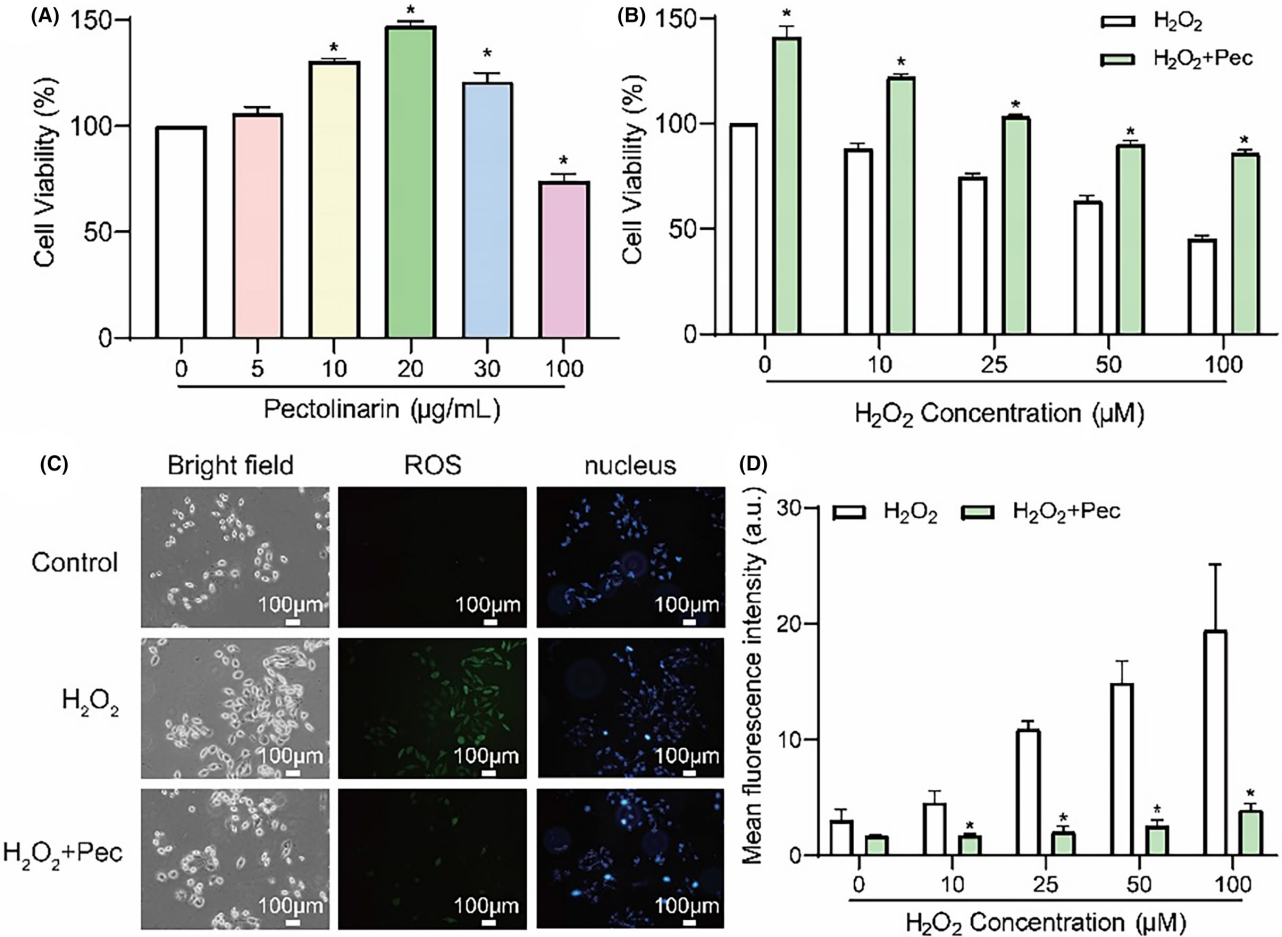
Evaluation of the antioxidant capacity of pectolinarin. (A) Cell viability at different concentrations of pectolinarin (0, 5, 10, 20, 30 and 100 μg/mL). *p < 0.05 when compared with 0 μg/mL. (B) Cell viability under the effect of different concentrations of H_2_O_2_ with (green) or without (white) 20 μg/mL of pectolinarin. (C) Fluorescence pictures of fibroblast ROS (green) under H_2_O_2_, Control, H_2_O_2_ + Pec, cell nucleus (blue) and bright field cell pictures. (D) Comparison of fluorescence intensity under H_2_O_2_ (white) and H_2_O_2_ + Pec(green). Pec, pectolinarin; ROS, reactive oxygen species.

To assess the impact of pectolinarin solutions could affect cell viability, we measured cell viability levels at different pectolinarin concentrations. Compared to the blank group, cell viability improved to approximately 145% at a pectolinarin concentration of 20 μg/mL (Figure 3A). However, cell viability significantly decreased at a concentration of 100 μg/mL, suggesting that 20 μg/mL may be the optimal pectolinarin concentration for effective cellular action. Subsequently, we investigated whether pectolinarin at a concentration of 20 μg/mL could effectively repair damage caused by ROS and act as an antioxidant. Fibroblasts were exposed to different H_2_O_2_ concentrations to induce excessive ROS production (Figure 3B), simulating cellular oxidative stress condition. The experimental group received a pectolinarin solution at a concentration of 20 μg/mL to evaluate its potential in reversing H_2_O_2_‐induced cell damage. The CCK8 assay demonstrated that the addition of 20 μg/mL of pectolinarin significantly reversed cell viability compared to the H_2_O_2_ group. Fluorescence images revealed that cells produced substantial ROS in the presence of H_2_O_2_, but the ROS level was significantly reduced with the addition of pectolinarin (Figure 3C). Analysis of the mean green fluorescence intensity in cells with and without pectolinarin under H_2_O_2_ treatment indicated that pectolinarin effectively mitigated the damage caused by H_2_O_2_ (Figure 3D).

### 3.4 Angiogenesis of PF‐127@P dressings

**FIGURE 4:**
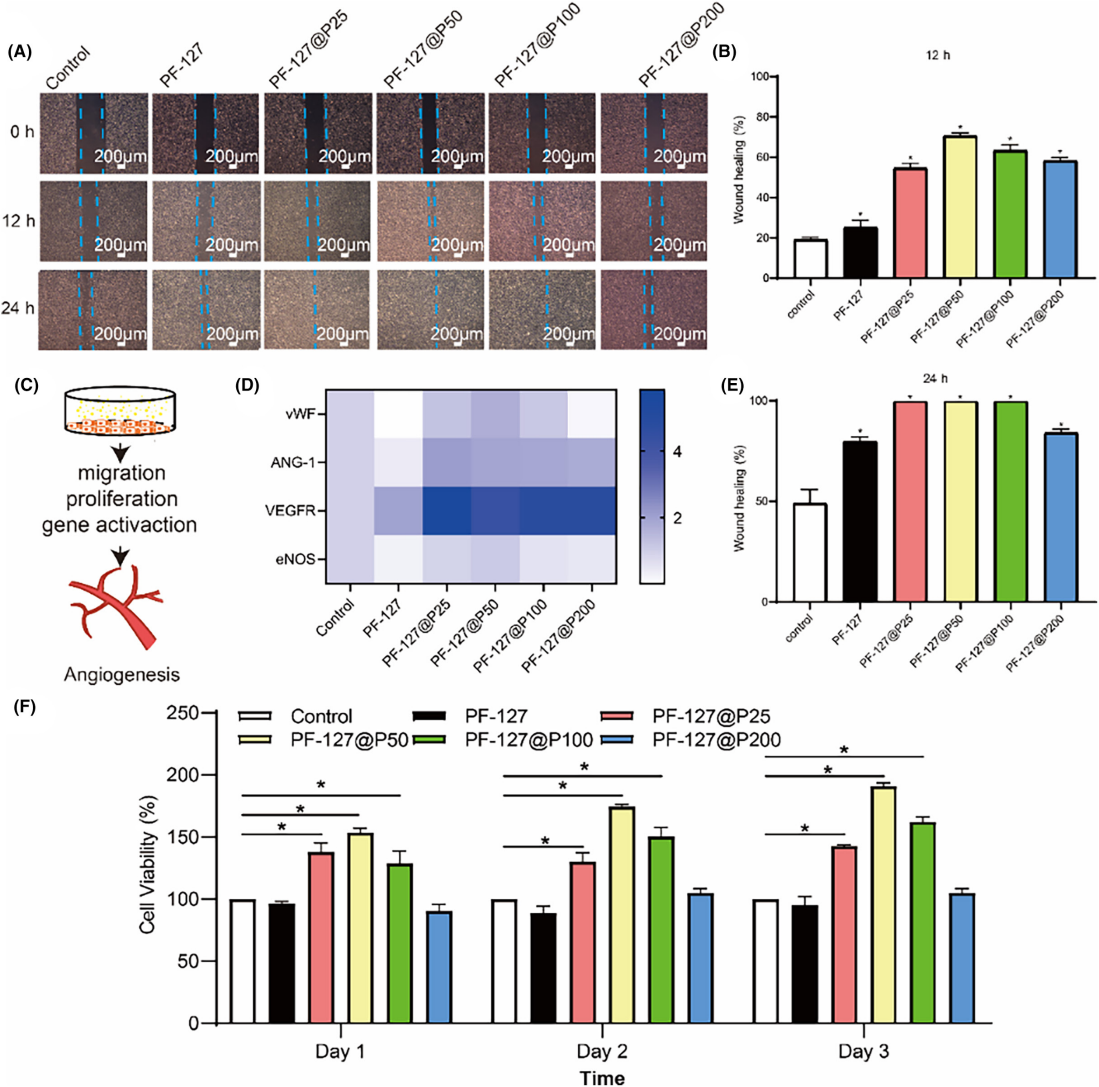
The proliferation, migration and gene activation of endothelial cells. (A) Migration images at 12 h and 24 h. (B, E) Statistical analysis of scratch healing images at 12 h and 24 h. (C) Schematics of pectolinarin promoting angiogenesis. (D) Expression of angiogenic genes. (F) Proliferation of endothelial cells (Days 1, 2 and 3). ANG‐1, Angiopoietin 1; eNOS, endothelial nitric oxide synthase; VEGFR, vascular endothelial growth factor receptor; vWF, von Willebrand factor. *p < 0.05 when compared with control.

Angiogenesis is a vital process for wound healing, providing nutrients, supporting local collagen synthesis and remodelling, and facilitating re‐epithelialization.^3^ The migration and proliferation of endothelial cells, along with the expression of angiogenesis‐related genes, are crucial for angiogenesis^41^ (Figure 4C).

#### 3.4.1 Proliferation

First, we assessed the ability of PF‐127@P dressings to promote endothelial cell proliferation using the CCK8 assay. Endothelial cells were inoculated in 96‐well plates, and PF‐127@P hydrogel extracts were used to intervene with the cells. On day 1, cells did not exhibit proliferation with PF‐127 hydrogel alone (Figure 4F). However, after adding pectolinarin, cell proliferation continued until the concentration exceeded 100 μg/mL. None of the concentrations showed toxic side effects on cells, indicating that pectolinarin promotes endothelial cell proliferation at a more suitable concentration.

#### 3.4.2 Migration

Endothelial cell migration iscrucial for angiogenesis and wound repair, making it a potential therapeutic target for regulating blood vessels.^42^, ^43^ We evaluated the migration ability of endothelial cells using the scratch assay. As shown in Figure 4, PF‐127 alone had a weak ability to promote endothelial cell migration. The PF‐127@P50 group exhibited a healing rate of approximately 75% at 12 h, significantly different from the control group (PBS group) (Figure 4A, B). At 24 h, all groups treated with pectolinarin showed complete healing, except for the PF‐127@P200 group (Figure 4A, E). These findings align with the results of cell proliferation, as a pectolinarin concentration of 200 μg/mL showed no significant promotion effect and exhibited cell viability similar to PF‐127 alone.

#### 3.4.3 Activation angiogenesis‐related genes

During angiogenesis, a balance between the activation of pro‐angiogenic genes and the inhibition of inhibitory factors stimulates angiogenesis.^44^ The von Willebrand factor (vWF) gene is involved in coagulation and assists in platelet adhesion, promoting haemostasis.^45^ As shown in Figure 4D, the expression of the vWF gene was elevated by more than 1.7‐fold in the PF‐127@P50 hydrogel extract group compared to the group without pectolinarin, and enhanced expression of the vWF gene was observed in all pectolinarin‐treated groups except for PF‐127 hydrogels alone. Angiopoietin 1 (ANG‐1) plays a role in vascular development and mediates the maturation and stabilization of blood vessels.^46^ The addition of pectolinarin increased the expression level of this gene. Vascular endothelial growth factor receptor (VEGFR) is a crucial protein in vascular development, cell mitosis and migration.^47^ The expression of this gene was significantly increased with the assistance of pectolinarin, particularly in the PF‐127@P25 group. Endothelial nitric oxide synthase (eNOS), also known as nitric oxide synthase 3 (NOS3), regulates cell proliferation and platelet adhesion.^48^ eNOS was upregulated only with the addition of pectolinarin at 50 μg/mL. By contrast, none of the above genes showed significant increases in the PF‐127 hydrogels group compared to the blank group. These results suggest that pectolinarin enhances the expression of angiogenesis‐related genes, and with pectolinarin‐loaded hydrogel plays a significant role in promoting angiogenesis during wound healing.

### 3.5 Promoting scarless wound healing in vivo

**FIGURE 5:**
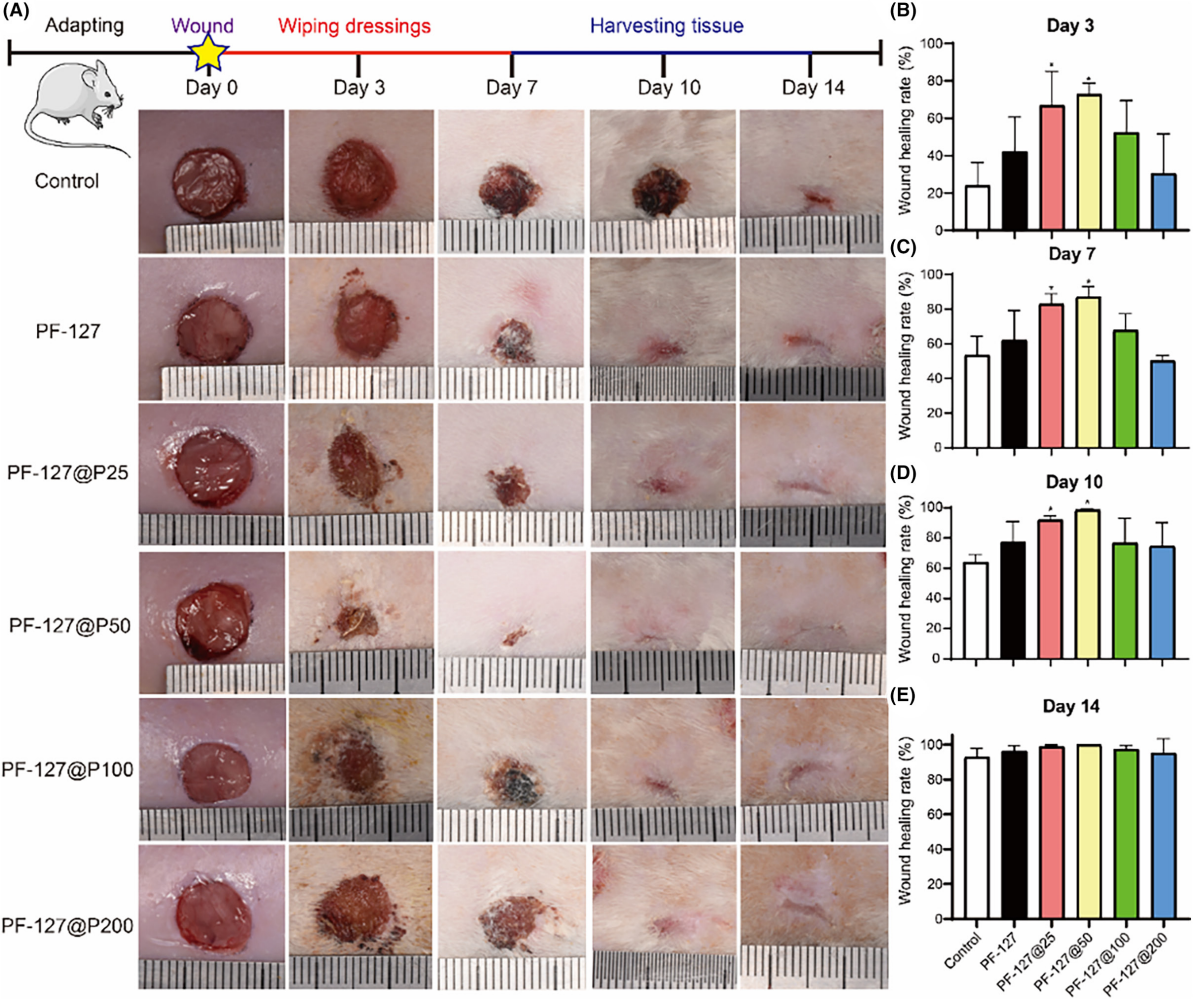
Dynamic process of wound healing under PF‐127@P dressings. (A) The dynamic process and schematic diagram of skin defect repair in rats with PF‐127@P hydrogels at Days 0, 3, 7, 10 and 14. (B–E) Wound healing rates at days 3, 7, 10 and 14. *p < 0.05 when compared with control.

In this study, we investigated the therapeutic effects of PF‐127@P dressings on wound healing using a rat skin defect model. The aim of our study was to determine the potential of local pectolinarin release in enhancing wound healing. As depicted in Figure 5A, the PF‐127@P50 group exhibited a healing rate of over 70% on day 3, with the PF‐127@P25 group also showing significantly higher healing rates compared to the control group. By day 7, the PF‐127@P50 group achieved a healing rate of 87%, consistently outperforming the other subgroups. However, the wound healing rate of the PF‐127@P200 group was lower than that of the control group on day 7, suggesting a possible negative effects of high concentrations of pectolinarin on wound healing. In the previous section, we discussed the effects of pectolinarin on cellular antioxidant capacity, proliferation, migration and angiogenesis. Figure 3A indicates that pectolinarin exhibited an inhibitory effect on cell viability at a concentration of 100 μg/mL. However, the precise amount of local pectolinarin released from the hydrogel in vivo remains undetermined.

**FIGURE 6:**
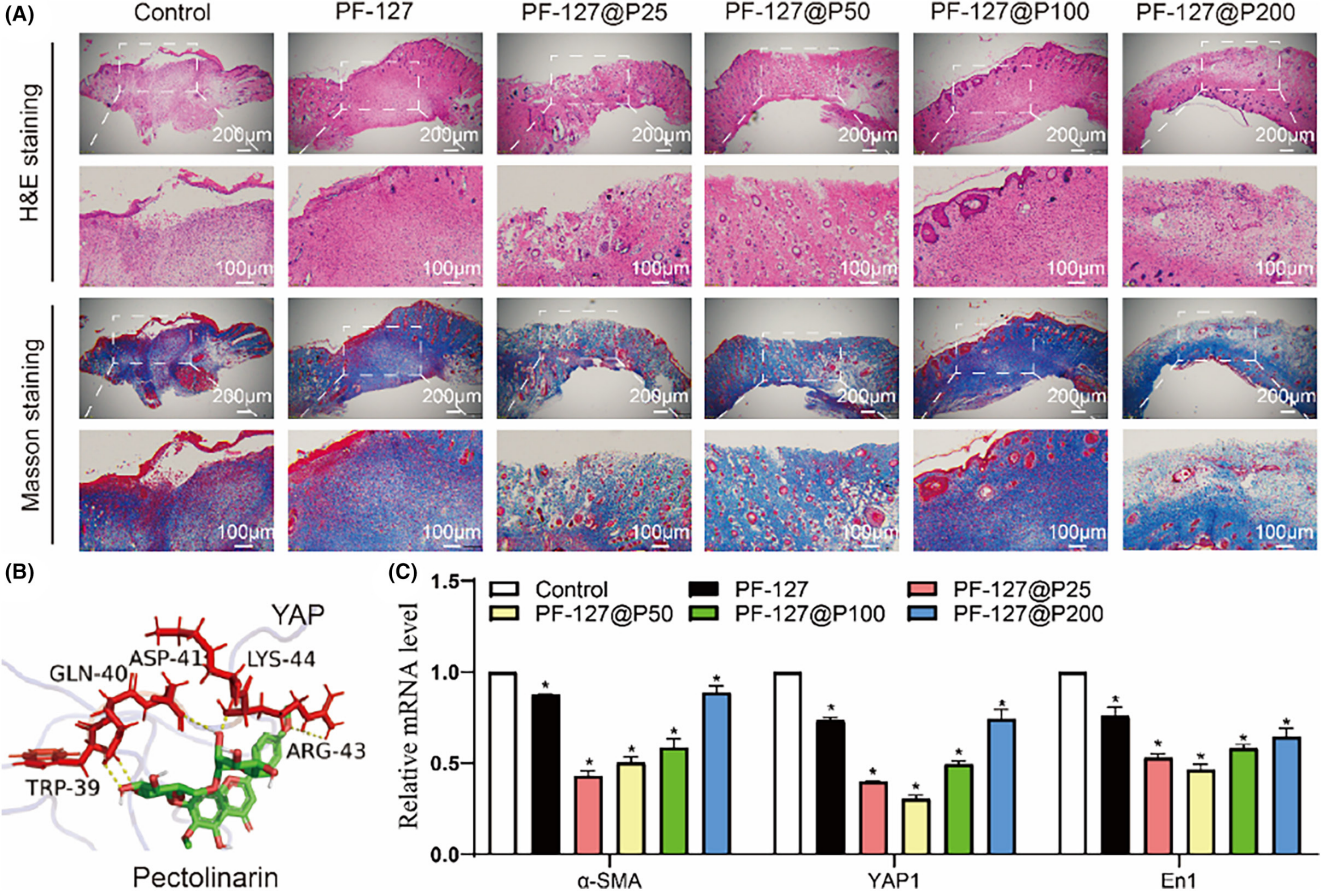
Wound healing image and the scarless regeneration mechanism. (A) Haematoxylin and eosin and Masson staining images of wound on day 14 under PF‐127@P dressing. (B) The results of molecular docking experiments of the action of pectolinarin and YAP. (Yellow is the hydrogen bond, red is the site of pectolinarin binding on YAP and green is pectolinarin.) (C) Expression of α‐SMA; YAP and En1. α‐SMA, α‐smooth muscle Actin; En1, Engrailed‐1; YAP, Yes1 associated transcriptional regulator. *p < 0.05 when compared with control.

In addition to promoting wound healing, PF‐127@P hydrogels significantly reduced scar formation. As demonstrated in Figure 5A, scars were notably reduced and collagen fibres appeared more aligned at a pectolinarin concentration of 50 μg/mL, with a small number of hair follicles and sebaceous glands visible, resembling normal skin tissue (Figure 6A). These findings align with the results of cellular experiments, except that the optimal concentration for enhancing wound healing in cellular tests couldn't be determined. Nonetheless, our in vivo experiments clearly demonstrated that PF‐127@P50 dressings ultimately promoted wound healing. This outcome may be attributed to a combination of factors, including PF‐127@P50 dressings' ability to promote endothelial cell proliferation, migration and angiogenic gene expression, as well as its antioxidant effect. Figure 1C indicates that the cumulative release of PF‐127@P50 dressings at 36 h was approximately 40 μg/mL, which is considered moderate and does not cause cytotoxicity due to local buildup.

Furthermore, we observed varying degrees of skin appendage regeneration and re‐epithelialization in all dressing groups with the addition of pectolinarin. While excessive pectolinarin did not accelerate wound healing, further investigation is needed to elucidate by which pectolinarin enhances hair follicle regeneration.

**FIGURE 7:**
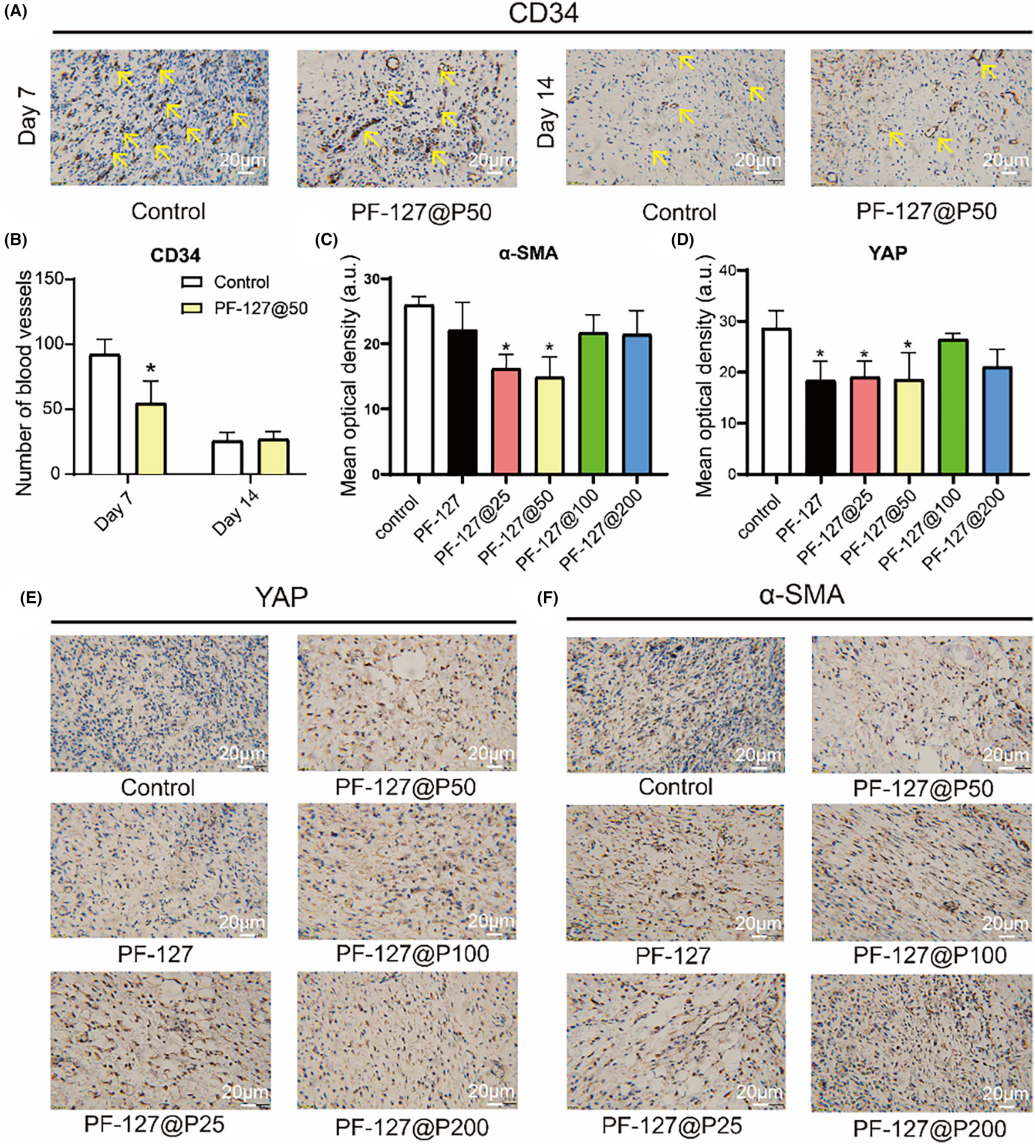
Immunohistochemistry (IHC) image of wound healing. (A) Expression of CD34 (yellow arrows represented CD34) in the Control and PF‐127@50 groups on days 7 and 14. (B) Immunohistochemical statistics of vessel number in the Control and PF‐127@50 groups on days 7 and 14. (C) Statistical plot of YAP expression in tissues at day 14. (D) Statistical plot of YAP expression in tissues at day 14. (E) Expression of YAP in tissues at day 14. (F) Expression of α‐SMA in tissues at day 14. *p < 0.05 when compared with control.

We also evaluated angiogenesis during the wound healing process in vivo. Granulation tissue thrives a moist environment to thrive, and PF‐127 hydrogel provides an ideal wound healing environment.^22^ Angiogenesis is critical for expediting wound healing, and once wound healing, and during the remodelling phase, which capillaries in the wound shrink.^49^ By day 7, the PF‐127@P50 hydrogel group exhibited fewer local blood vessels than the control group, while on day 14, there was no significant difference between the two groups (Figure 7A, B). This suggests that capillaries in the PF‐127@P50 hydrogel group formed and underwent alterations earlier than those in the control group. It is worth noting that at present there is still a lack of gold standard controlled protocols for scar‐free regeneration, although many studies have shown promising results against scar production.

### 3.6 Pectolinarin's potential in promoting scarless wound healing through YAP gene expression inhibition

Wound healing aims to restore disrupted tissue; however, external factors like infection and ROS can lead to excessive fibrosis and undesired scar tissue.^50^ Pectolinarin has been reported to possess anti‐ROS, anti‐inflammatory and antibacterial properties.^51^, ^52^ However, recent studies have highlighted the role of the En1 gene in fibroblasts, regulated by the YAP gene, in scar formation.^14^, ^53^ Scar formation is also influenced by the local skin tension,^54^, ^55^ which can be reduced with the support and adherence of hydrogel dressings.^56^ While scar tissue was still observed in the PF‐127 group, it nearly disappeared in the PF‐127@P50 group, indicating pectolinarin's inhibitory effect on YAP gene expression may be primarily responsible for the scarless regeneration effect.

Although there is empirical and anecdotal evidence supporting the use of Cirsium japonicum in heal burns and scalds and promoting scarless regeneration, the underlying mechanisms remain poorly studied. We employed a network pharmacology approach to preliminarily elucidate the potential mechanism of action and discovered that pectolinarin might play a key component in inhibiting scar production (unpublished). Subsequently, we conducted molecular docking experiments^15^ to simulate the binding of pectolinarin and YAP and observed that they formed five hydrogen bonds with a binding energy of −7.7, indicating strong binding affinity (Figure 6B). PCR results demonstrated varying degrees of YAP and its downstream gene En1 inhibition in all PF‐127 groups (Figure 6C). Pectolinarin also suppressed the expression of α‐smooth muscle actin (α‐SMA), which is closely associated with scar formation.^57^ Immunohistochemistry results further suggested that the significant downregulation of α‐SMA and YAP expression in the PF‐127@P25, PF‐127@P50 and PF‐127@P200 groups (Figure 7C–F), confirming pectolinarin's inhibitory effect on scar‐related gene YAP.

While the dressing may have additional functions, such as inhibiting collagen deposition or disrupting bacterial growth to create a more favourable healing microenvironment, our findings suggest that the inhibition of YAP gene expression by pectolinarin plays a crucial factor in promoting scarless wound healing. In future studies, we plan to investigate the specific regulatory mechanisms between pectolinarin and YAP genes, as well as their potential role in encouraging hair follicle regeneration.

## 4 CONCLUSION

The multifunctional temperature‐sensitive PF‐127@P dressing was successfully developed as a platform for delivering pectolinarin, enabling the inhibition of YAP and the promotion of scarless wound healing. The dressing released bioactive pectolinarin, which facilitated endothelial cell proliferation, migration and activation of vascular‐related genes. Additionally, pectolinarin exhibited significant antioxidant activity and reversed cell damage caused by ROS. Application of the dressings to the wounds demonstrated favourable biocompatibility and resulted in higher wound healing rates at loading levels of 25, 50 and 100 μg/mL, surpassing unfilled defects or PF‐127 hydrogels alone. Furthermore, in a full skin defect model, the adding 50 μg/mL pectolinarin to the dressings demonstrated the most effective promotion of wound healing and reduction of scar formation. This group also displayed intact skin tissue, including hair follicles and sebaceous glands, as well as well‐aligned collagen fibres. The combination of pectolinarin, a natural flavonoid, with PF‐127 hydrogels represents a simple yet effective strategy for scar‐free skin healing in future clinical applications.

## AUTHOR CONTRIBUTIONS

**Xiaohang Chen:** Conceptualization (lead); methodology (lead); visualization (lead); writing – original draft (equal); writing – review and editing (equal). **Haoyue Song:** Conceptualization (equal); methodology (equal); supervision (equal); writing – review and editing (equal). **Kun Song:** Visualization (supporting); writing – review and editing (supporting). **Yuan Zhang:** Supervision (equal); writing – review and editing (equal). **Jia Wang:** Supervision (supporting); writing – review and editing (supporting). **Jinjia Hong:** Supervision (supporting); writing – review and editing (supporting). **Qingpeng Xie:** Supervision (supporting); writing – review and editing (supporting). **Jing Zhao:** Supervision (supporting); writing – review and editing (supporting). **Meixian Liu:** Supervision (supporting); writing – review and editing (supporting). **Xing Wang:** Conceptualization (supporting); funding acquisition (equal); supervision (supporting); visualization (supporting); writing – review and editing (equal).

## FUNDING INFORMATION

This work was supported by the National Natural Science Foundation of China (82071155, 82271023 and 82301052), Shanxi Applied Basic Research Program Outstanding Youth Cultivation Project Fund (202203021223006), the Shanxi Applied Basic Research Program Science‐Youth Technology Research Fund (20210302124398), the Scientific and Technological Innovation Programs of Higher Education Institutions in Shanxi (2020 L0209), and the Open Project of Shanxi Province Key Laboratory of Oral Diseases Prevention and New Materials (KF2020‐07).

## CONFLICT OF INTEREST STATEMENT

The authors declare that the research was conducted in the absence of any commercial or financial relationships that could be construed as a potential conflict of interest.

## Supporting information


Figure S1

## Data Availability

The data that support the findings of this study are available on request from the corresponding author. The data are not publicly available due to privacy or ethical restrictions.
