# Supplementary figures and images for: Temperature‐sensitive hydrogel releasing pectolinarin facilitate scarless wound healing
Source: J Cell Mol Med. 2024 Feb 8;28(4):e18130. doi: 10.1111/jcmm.18130 (PMC10853586; doi:10.1111/jcmm.18130)

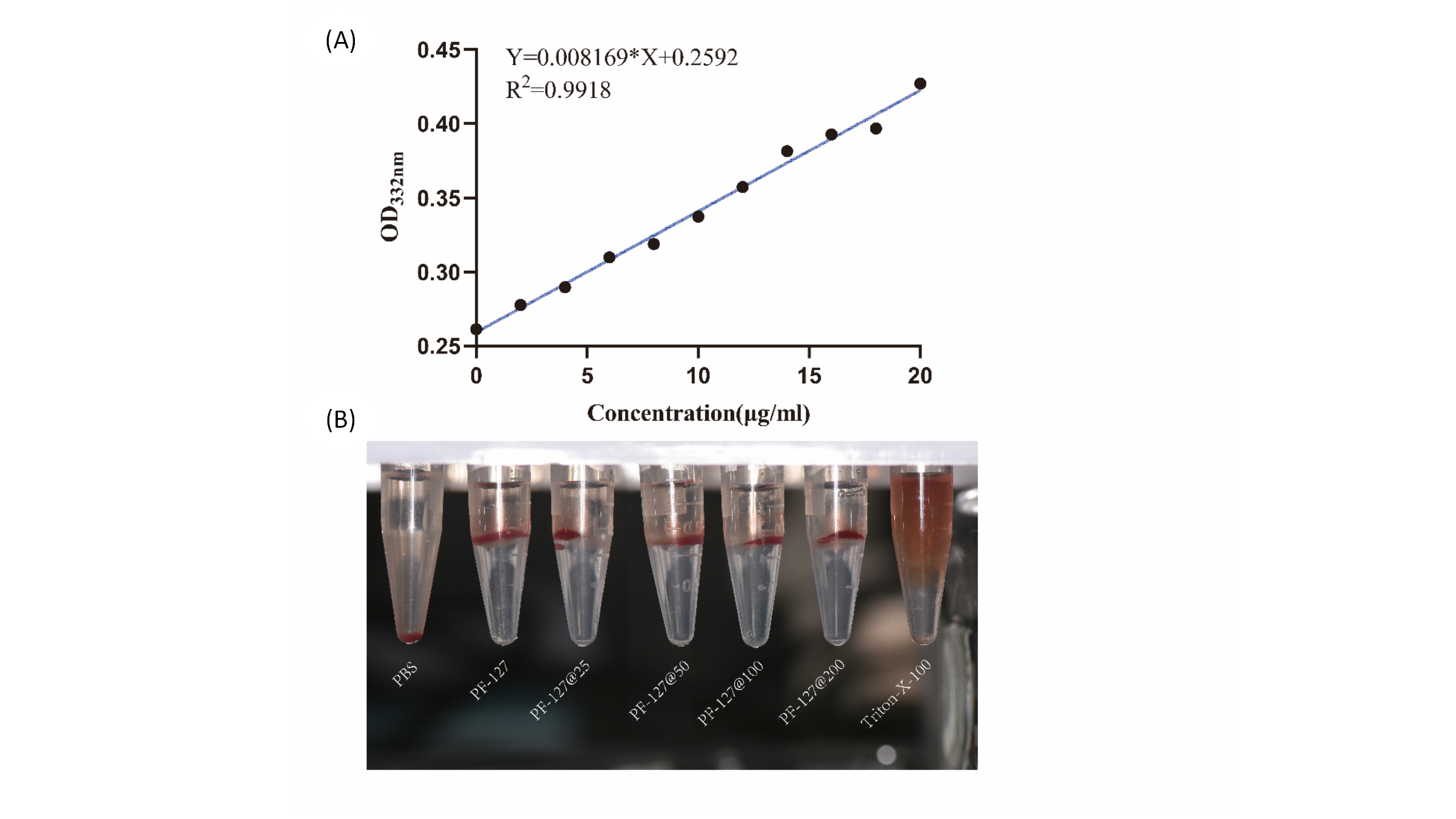


**Supplementary figure 1.** (A) Standard curve of pectolinarin; (B) Visualization of hemolysis assay.

Supplement: Supplementary file 1 — Figure S1 [file JCMM-28-e18130-s001.docx]
